# Assessment of Rosemary (*Rosmarinus officinalis* L.) Extract as Antioxidant in Jelly Candies Made with Fructan Fibres and Stevia

**DOI:** 10.3390/antiox9121289

**Published:** 2020-12-16

**Authors:** Cristina Cedeño-Pinos, Magdalena Martínez-Tomé, María Antonia Murcia, María José Jordán, Sancho Bañón

**Affiliations:** 1 Department of Food Technology and Science and Nutrition, Veterinary Faculty, Regional Campus of International Excellence “Campus Mare Nostrum”, University of Murcia, 30100 Murcia, Spain; cristinacarmen.cedenop@um.es (C.C.-P.), mmtome@um.es (M.M.-T.), mamurcia@um.es (M.A.M.); 2 CIBER: CB12/03/30038 Pathophysiology of Obesity and Nutrition, CIBERobn, Carlos III Health Institute (ISCIII), 28013 Madrid, Spain; 3 Research Group on Rainfed Crops for the Rural Development, Murcia Institute of Agri-Food Research and Development (IMIDA), c/Mayor s/n, 30150 La Alberca Murcia, Spain; mariaj.jordan@carm.es

**Keywords:** rosemary, polyphenols, rosmarinic acid, antioxidant, sugary products

## Abstract

Enrichment with rosemary antioxidants is proposed as a possible strategy to obtain healthier jelly candies. Two aqueous rosemary extracts (RE) containing 73.9 (RE74) and 145.6 (RE146) mg polyphenols per g fresh weight were assessed as antioxidants in jelly candies based on fructooligosaccharides, inulin and stevia. Up to 15 phenolic acids, flavonoids and diterpenes were determined in the extracts, with rosmarinic acid as the main active compound. Sensory tolerance, physical properties, rosmarinic acid recovery, polyphenol content, and antioxidant capacity were determined in jelly candies. The threshold of sensory detection was established at 0.26 g RE146/kg of raw candy, below which rosemary off-flavours were avoided without altering pH, brix, texture, CIELab colour, and consumer acceptance. Adding 0.26 g RE146 per kg increased (*p* < 0.001) polyphenol content from 197 to 411 µg GAE/g and the antioxidant capacity from 1.77 to 4.14 μmol Trolox/g. Rosemary polyphenols resulted in being resistant to cooking, acted as secondary antioxidants and showed good interaction with the other jelly ingredients. Aqueous extracts from rosemary distillation by-products can be incorporated at acceptable levels to jelly candy formulations leading to higher oxidative stability and an increased content of dietary polyphenols.

## 1. Introduction

Sugary candy healthiness is being questioned due to its high content in assimilable carbohydrates and low content in fibres, proteins, vitamins or antioxidants. In recent years, the Spanish Government has introduced several legal restrictions concerning candy sale to the child population [1]; furthermore, the implementation of nutritional labels (e.g., Nutriscore) in the European Union negatively computes products rich in calories and sugar [2]. Aware of this problem, the confectionary industry is making efforts to develop new products with better nutritional properties that will meet the demands of consumers regarding a healthier diet [3]. The most common strategies used in improving candy formulations involve replacing sugars with sweeteners [4,5]; the substitution of azo dyes for natural ones [6,7]; or the incorporation of dietary fibre, vitamins, fruit derivatives, and plant extracts [7,8,9,10]. Regarding the latter, different studies on candies made with phenolic extracts of tea [11], bougainvillea [12], mint and chamomile [13] revealed their potential for inclusion as antioxidants or nutraceutical ingredients in candies. However, the application of such plant extracts in candies is still little assessed despite the wide range of extracts available.

Rosemary (*Rosmarinus officinalis* L.) is considered a natural source of phenolic compounds. Rosemary essential oils mainly contain volatile phenols (e.g., eucalyptol, pinene and carvacrol), while oil-free leaves contain mainly non-volatile polyphenols (e.g., rosmarinic and carnosic acids) that provide a lower off-flavour to rosemary in food matrices [14]. At present, RE are authorized by the European Union for use as a natural antioxidant in meat and fish products [15]. The effectiveness of RE as an antioxidant has been demonstrated in several meat products [16,17] and in some vegetable products such as biscuits [18], although their use in candies has barely been explored. In the case of RE, the antioxidant and antimicrobial activities mainly rely on its phenolic acid, flavonoid and diterpene contents [19], whose levels may vary depending on the extraction procedure used [20] as well as agronomical factors affecting the plant material (environment, abiotic stress, genetic inheritance and maturation stage) [21,22]. As a consequence, rosemary plants from different origins may provide REs with different antioxidant and sensory properties (browning, rosemary notes, bitterness, astringency, etc.) when applied in food matrixes.

Jelly candies are widely consumed, especially by children and adolescents due to their soft and elastic texture and wide variety of fruity flavours [13]. They are basically aqueous dispersions made with sugars, gelatine, starch, pectin, acids, and other minor ingredients obtained under moderate thermal treatments, a fact which offers good opportunities for enrichment with active compounds [3,6,23]. Previous research [9] showed good properties of fructan fibres (chicory inulin and fructooligosaccharides) as alternative ingredients for replacing starches and sugars in jelly candy formulations. In addition, REs can be used to make jelly candies enriched with antioxidants. Among these, the REs containing hydrophilic polyphenols (e.g., rosmarinic acid) may be suitable for aqueous food matrixes of low pH, such as jelly candies, which favour the dissolution and stabilization of phenolic acids, enhancing their antioxidant properties [24]. Regardless of legal aspects, a technological approach including sensory tolerance, polyphenol stability and antioxidant properties, is required so these REs can be properly incorporated to jelly candy formulations. The threshold of sensory detection for REs should be established in jelly candies to attempt reaching the maximum antioxidant activities without affecting consumer acceptance. The resulting oxidant–antioxidant balance may be affected by several factors such as the polyphenol amount of REs, their degradation under heat-acidic condition, or the interactions existing among rosemary polyphenols and other candy ingredients. A same RE might present different antioxidant activities in jelly candies based on sweeteners and fructan fibres compared to sugary matrixes. The objective of the present study was to assess two aqueous REs (of low and high polyphenol content) obtained from distillation by-products as antioxidants in a jelly candy made with fructan fibres and stevia.

## 2. Materials and Methods

### 2.1. Experimental Design

Two aqueous REs of low (RE74) or high (RE146) polyphenol content were added at four different levels (0, 0.13, 0.26 and 0.52 g/kg raw jelly) to a jelly candy made with fructan fibres and sweeteners. Sensory tolerance, physical properties, rosmarinic acid recovery, polyphenol content, and antioxidant capacity were determined, following a randomized statistical design. A two-factor analysis of variance was performed to determine the effects of treatments (RE type and dosage) on the dependent variables. A Tukey test was used to compare the group means (*p* < 0.05). A different number of candy units (from 18 to 200) from at least three different manufacturing batches were sampled depending on the analysis to be performed. The software used was Statistix 8 package for Windows (Analytical Software, New York, NY, USA).

### 2.2. Rosemary Extracts

Two extracts were prepared with fresh rosemary leaves from plants belonging to the experimental farm of Murcia Institute of Agri-Food Research and Development, located in La Alberca, Murcia, Spain (IMIDA). Knowing the chemical intraspecific variability that this *Labiateae* exhibits [21], the richness in the polyphenolic fraction was the base for the selection of the rosemary plants.

Firstly, and in order to remove the essential oil content, collected leaves were dried by forced-air at 35 °C for 48 h until constant weight and then distilled in a water steam at 100 °C for 3 h using a Clevenger-type apparatus. A second solid/liquid extraction was performed to obtain the rosemary Extracts (RE). The distilled leaves were ground to 2 mm and the resulting powder was mixed with deionized water at 1:10 ratio (*w*:*v*) and kept at 30 °C under constant stirring for 90 min. The mixture was centrifuged at 5000 rpm and 5 °C for 10 min and the resulting supernatant was filtered through Whatman filter paper (No. 4), lyophilized at 100 mbar and −80 °C for 24 h (Lyobeta 15, Telstar) and stored in a dark steel container at −80 °C until later use.

RE polyphenolic profile was determined by HPLC-DAD, as described by Jordán et al. (2013). The extracts were filtered at 25 µm (Millipore SAS, Molsheim, France), and 20 µL were injected into an HPLC-1200 Series (Agilent, Waldbronn, Germany) equipped with a G1311A binary pump and a G1315A photodiode array UV/Vis detector. A Zorbax SB-C18 reverse phase column was used (4.6 × 250 mm, pore size 0.25 µm), preceded by a (Zorbax SB-C18 pre-column (4.6 × 125 mm, pore size 0.25 µm), both from Agilent Technologies, USA. As mobile phase, acidified water (formic acid 0.05%) was used in channel A, and 100% acetonitrile (Fisher Chemical Spain) in channel B. The gradient was as follows: 0 min, 5% of B; 10 min, 15% of B; 30 min, 25% of BA; 35 min, 30% of BA; 50 min, 55% of BA; 55 min, 90% of B; 70 min, and 100% B, which was maintained for 10 min before returning to the initial conditions. The flow rate was 1.0 mL/min and the detection wavelengths were set at 280 and 330 nm. The standards were provided by (i) Sigma-Aldrich: Luteolin -7-O-β-Glucoronide (CAS 29741-10-4); Cirsimaritin (CAS 6601-62-3); Genkwanin (CAS 437-64-9); Hesperidin (CAS 520-56-3); Luteolin (CAS 491-70-3); 7-methyl-rosmanol (CAS 113085-62-4); Carnosol (CAS 5917-80-2); Carnosic acid (CAS 3650-09-7); 12-O-methylcarnosic acid (CAS 62201-71-2); (ii) Acros Organics: Ferulic acid (CAS 1135-24); Caffeic acid (CAS 331-39-5); (iii) Fluorochem: Salvianic acid (CAS 76822-21-4); (iv) Fluka: Rosmarinic acid (CAS 20283-92-5); and Luteolin-7-glucoside (CAS 5373-1135). Identification was made by comparing retention times with the respective spectra, and quantification was carried out with linear regression models of standard dilutions. The results were expressed as mg/g.

Calibration curves were developed from the HPLC-DAD using specific standards for each polyphenol compound. Eight increasing concentrations were used at different ranges depending on the polyphenol compound (overall, from regression 0.03 to 600 µg/mL). The limits of detection (LOD) and quantification were determined using the average of the standard deviation of the response (SD) and the slope of the calibration curve (S) according to the formulas LOD = 3.3(SD / S), LOQ = 10(SD / S).

### 2.3. Jelly Candy Manufacturing

Jelly candies were made as described by [9] with some modifications. The raw ingredients (g/kg) were: fructooligosaccharides (FOS) (705.8); chicory inulin (114.4); water (110.8); pork gelatine type A (41); citric acid (12); lactic acid (6); strawberry flavour (3); *Stevia rebaudiana* powder (1.4); sodium citrate (1); carminic acid (0.5); and rosemary extract (0–0.52). The type A pork gelatine was provided by Juncà Gelatines, SLU (Barcelona, Spain), while the FOS water solution (Fosvitae chemical synthesis 72 °Brix) and stevia powder were provided by Zukán (Molina de Segura, Murcia). Chicory inulin (Orafti^®^ GR) was provided by Beneo-Orafti, Belgium. The pork gelatine was dissolved in hot (80 °C) water (2:1 *w*:*w*) while stirring for 30 min. Inulin was mixed with the FOS solution and homogenized with an Ultraturrax (11500 rpm) at room temperature for 5 min until a cream was obtained. The inulin cream and gelatine solution were mixed at 80 °C under stirring for 10 min in a kitchen robot Mycook (Taurus, Lérida, Spain). Once homogenized, the remaining ingredients (including the RE) were added and the mixture was homogenized for 10 min. The jelly mass was immediately placed in trays containing cylindrical moulds (26 mm diameter and 9 mm deep) printed on corn starch (pre-conditioned at 30 °C and 10% RH for 24 h) and dried in a climatic chamber (Climacell 707 (MMM Medcenter Einrichtun-gen GmbH, Munchen, Germany) with circulating air (at 25 °C and 30% RH) for 24 h. Subsequently, the jellies were demoulded, waxed with carnauba wax and kept in polypropylene bags and darkness at 25 °C for 7 days until analysis.

### 2.4. Sensory Quantitative Descriptive Analysis (QDA)

A sensory descriptive analysis [25] was performed to assess jelly candy flavour focusing on rosemary off-flavours. Fifteen panellists selected from Murcia University and IMIDA personnel were specifically trained during four sessions according to [26]. The first two training sessions were concerned with identifying, selecting and quantifying the flavour attributes. After reaching a consensus, six jelly candy flavour descriptors were chosen: strawberry, sweetness, acid, bitter, astringent, and rosemary (rosemary-herbal and pungent). The descriptors were quantified using intensity scales graduated in one-point intervals: 1: absent; 2: slight; 3: moderate; 4: intense; 5: very intense. Six water solutions of different concentrations (g/kg) were used to establish these scales: strawberry (strawberry aroma from 5 to 25), sweetness (sucrose from 40 to 200), acid (citric acid from 0.8 to 4), bitter (stevia powder from 0.13 to 0.65), astringent and rosemary-herbal (RE from 0.03 to 0.15). The following two training sessions were concerned with quantifying the flavour attributes in jellies with different added quantities of RE (see experimental design). Four randomly coded samples were assessed in triplicate by each panellist in each session. The results were expressed as sensory score.

### 2.5. Triangular Sensory Test with Consumers

The QDA results were contrasted in a further sensory study of jelly candy flavour with consumers. Two (RE74 and RE146) triangular trials [27] were conducted at the University of Murcia and IMIDA to discriminate candy favour. The panel was composed of 200 consumers, including staff from the two centres and visitors (145 women and 55 men, aged between 18 and 63 years old), who claimed they consumed jelly candies ranging from several times a month to several times a year. Two triangular tests on randomly coded samples were carried out with each consumer in each session.

### 2.6. Physical-Chemical Measurements

The pH was determined by dissolving 1 g of sample in 10 mL of water (50 °C) using a pH meter Crison model 2001 (Barcelona, Spain) equipped with a combined electrode, Cat. No. 52-22 (Ingold Electrodes, Wilmington, USA). The total soluble solids (g/100 g) were determined using an Atago manual digital refractometer (Pocket PAL-3, Co. Ltd., Tokyo, Japan). For the measurements, 2 mm thick slices of the sample were placed in the refractometer visor. The moisture content (g/100) was determined after dehydration using a D6450 drying oven (Heraeus, Boadilla del Monte, Madrid, Spain) and a BP 110S (0.001 g precision) scale (Sartorius, Alcobendas, Madrid, Spain) (Association of Official Agricultural Chemists AOAC, No 945.15, 2000) [28]. The ash content (g/100) was determined after mineralization at 550 °C for 24 h using an HK-11 muffle furnace (Forns Hobersal, Caldes de Montbui, Barcelona, Spain) (AOAC, No 923.03, 2000) [28]. Texture Profile Analysis (TPA) was performed using a QTS-25 Texture Analyser (Brookfield Engineering, Harlow, Essex, UK). The testing conditions were: 24 °C; TA3/100 flat cylindrical probe (20 mm in diameter); trigger point, 0.05 N; compression objective, 5 mm; cross-head speed, 0.1 mm/s; and charge cell, 10 kg. The texture variables analysed were: (i) Hardness (N), the force required to compress the material by a given amount; (ii) Cohesiveness (no units), strength of the internal bonds in the sample, the value being the ratio of the areas (force x time) resulting from the second and first bites; (iii) Springiness (mm), the elasticity recovered when the compressive force is removed, calculated as sample height recovered during the time elapsed from the end of the first bite to the beginning of the second; (iv) Chewiness (N.mm), energy required to chew a solid food into a state ready for swallowing, the value being the result of multiplying hardness x cohesiveness x springiness. Adhesiveness was not included since the jelly candies were lubricated with carnauba wax. Instrumental colour was measured on the candy surface by reflectance using a CR-200/08 Chroma Meter II (Minolta Ltd., Milton Keynes, UK) with a D65 illumination standard, 2° observer angle, and aperture size of 50 mm. The results were expressed as CIE units: lightness (L*), redness (a*) and yellowness (b*). All the physical measurements were made at least in triplicate.

### 2.7. Determination of Rosmarinic Acid

The remaining content of rosmarinic acid (the most abundant polyphenol present in both aqueous REs) was assessed in jelly candies. To determine its recovery in candies, a 3 g sample was melted at 60 °C and dissolved in methanol in a volumetric flask (10 mL). The solution was stirred for 10 min at 30 °C and then centrifuged at 3500 rpm and 5 °C for 10 min (D2010, Kubota, Japan). The resulting supernatant was collected and again centrifuged in an Eppendorf flask at 8000 rpm and 5 °C for 10 min (D-37520 Biofuge Pico centrifuge, Heraeus, Germany). The final supernatant was filtered at 0.22 µm and analysed by HPLC-DAD (see working conditions in rosemary extract subsection). Method accuracy was determined according to the guidelines of the International Conference of Harmonisation [29], by calculating polyphenol recovery after analysing candies containing known quantities of rosmarinic acid. Standard addition was done in triplicate at three concentration levels (1, 10 and 25 mg/mL). The percentage of recovery was calculated as the concentration recovered divided by the concentrations initially added and final results multiplied by 100.

### 2.8. Determination of Total Polyphenol Content

The total polyphenol content was determined in candies and their ingredients separately using the Folin–Ciocalteu method, for which 5 g samples were dissolved in a volumetric flask (10 mL) with methanol and centrifuged at 8000 rpm and 5 °C for 10 min. The supernatant was collected and frozen at −80 °C until analysis. Then, 5 mL of distilled water, 250 µL of sample solution and 800 µL Folin-Ciocalteu reagent were transferred to a 10 mL volumetric flask under stirring. After 8 min, 1.2 mL of 20% sodium carbonate (*v*/*v*) was added to the sample mixture, which was completed with distilled water and kept in a water bath (20 °C) for 2 h. Finally, sample absorbance was measured at 760 nm wavelength using a KNK-029-757 UV-visible spectrophotometer (Shimadzu, Duisburg, Germany). A calibration line was made using gallic acid as a standard. The results were expressed as µg gallic acid equivalents (GAE) per g sample.

### 2.9. Deoxyribose Damage

The products of the OH attack on deoxyribose were measured at 532 nm, as described by [30], with slight modifications. A 5 g sample was dissolved in 50 mL of Mili-Q water at 50 °C for 3 min under stirring to obtain sample solution. The widely used antioxidant additive, propyl gallate (PG), was also analysed at a concentration of 100 µg/g. The reaction mixture was prepared in a final volume of 1.2 mL by adding 100 µM EDTA (ethylenediaminetetraacetic acid), 50 µM FeCl_3_, 2.8 mM deoxyribose (if used), 2.8 mM H_2_O_2_, 25 µL in the case of jelly candy solution and 100 µL in the case of individual ingredient solutions (or 100 µL of the common food additives dissolved in water), and 10 mM KH_2_PO_4_-KOH buffer (pH 7.4). Ascorbate (100 µM) was added to start the reaction, and the tubes were incubated at 37 °C for 1 h. The results were expressed as the percentage of inhibition of the deoxyribose attack, where 100% attack is defined as the absorbance level recorded for deoxyribose without the addition of the tested compounds (control).

### 2.10. Antioxidant Capacity of Trolox Equivalents (TEAC)

The TEAC test was applied using the method described by [30], with slight modifications. The TEAC test measures the ability of antioxidants to extinguish a radical cation, 2.20-azino-bis-(3-ethylbenzothiazoline-6-sulfonic) (ABTS ±), in lipophilic and hydrophilic environments. The ABTS ± radical solution was generated from 2.5 mM ABAP (2,2′-azobis-(2-amidinopropane) hydrochloride) stock solution and 20 mM ABTS stock solution in phosphate buffer solution containing 100 mM phosphate and 150 mM NaCl at pH 7.4. These were incubated in darkness at 60 °C for 12 min and stored at room temperature. The absorbance at 734 nm was measured to check the formation of ABTS ± (absorbance between 0.35 and 0.45). The antioxidant activity of the samples tested (40 mL and 1960 mL of radical solution) was measured at 734 nm after 6 min and again after 24 h. A calibration curve was prepared with different concentrations of Trolox (standard solution was used to evaluate the equivalent antioxidant capacity, similar to water-soluble vitamin E).

## 3. Results

A total of 15 polyphenols were determined in RE: four phenolic acids (salvianic, caffeic, ferulic and rosmarinic), seven flavonoids (luteolin, luteolin-7-O-glucoside, luteolin-7-O-glucoronide, luteolin-7-glucoside derivate, cirsimaritin, genkwanin and hesperidin) and four diterpenes (carnosol, carnosic acid, 7-methyl rosmanol and 2-O-mehtyl-carnosic acid) (Table 1). Differences among the qualitative and quantitative polyphenolic profile detected among the rosemary extracts are related to the chemical variability exhibited by rosemary plants coming from the wild [21]. In this way, some compounds such as caffeic acid, ferulic acid, 7-methyl-rosmanol, and 2-O-mehtyl-carnosic acid were not detected in RE146, while luteolin and luteolin-7-O-glucoronide were not detected in RE74. Rosmarinic acid was the most abundant polyphenol quantified in both extracts (35.1 and 76.8 mg/g respectively) showing (RE146 a higher concentration of polyphenols (145.6 mg/g) than RE74 (73.9 mg/g), therefore, the RE146 would be expected to have a better antioxidant potential than the RE74.

The results of the QDA are shown in Table 2. RE addition did not affect (*p* > 0.05) the strawberry, sweet, acid or bitter flavour scores but did contribute some off-flavours. Astringency only scored higher (*p* < 0.01) in the medium-RE146 and high-RE146 candies, while rosemary (rosemary and pungent) flavour scored higher (*p* < 0.001) in all the RE candies than the untreated candies at medium and high doses. The threshold detection level for both RE74 and RE146 was reached at the medium dose (0.26 g RE/kg raw candy), which corresponded to 19.2 and 37.9 mg polyphenols per kg raw candy, respectively. Therefore, the presence of rosemary-herbal and pungent off-flavours in candies was probably more related to the quantity of added RE than with its polyphenol concentration. Other rosemary compounds, which are not polyphenols (e.g., tannins or essential oil residues), might have some sensory activity in strawberry jelly candies despite being made with flavouring agents mainly based on organic esters [31]. The results of the triangle discriminant test made with consumers are shown in Table 3. Consumers made between 30 and 38 correct identifications of the low- and medium-ER candies, respectively. According to the standard [27], at least 42 correct answers are required for flavour differences between treatments to be considered significant (n = 100; *p* < 0.05). Thus, an addition level of 0.26 g RE/kg raw candy was not perceived by the consumers.

**Figure 1:**
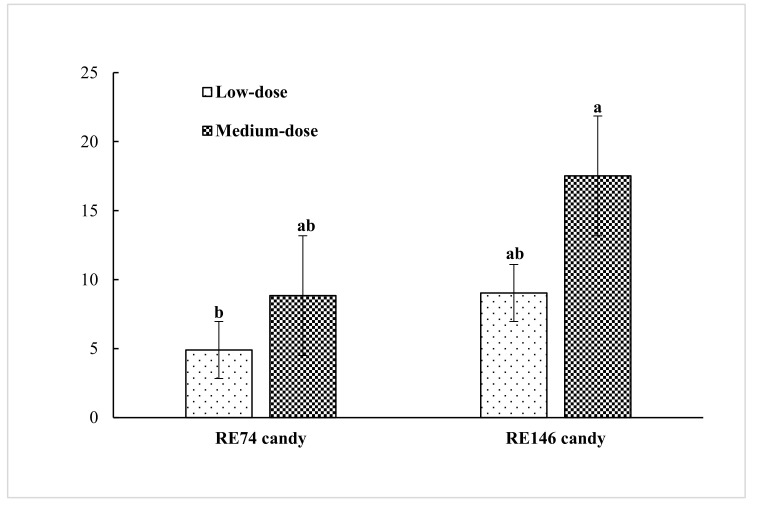
Rosmarinic acid recovery (μg/g) in jelly candies. Abbreviations: RE74 and RE146: rosemary extract of different polyphenol content. ^a, ab, b^ RE x D effects for *p* < 0.05.

The rosmarinic acid concentrations (μg/g) determined after jelly candy processing were: 4.9 (low-RE74), 9.0 (medium-RE74), 8.8 (low-RE146), and 17.5 (medium-RE146) (Figure 1). The quantities of rosmarinic acid (μg/g raw candy) added with the extracts were 4.5 (low-RE74), 9.1 (medium-RE74), 10.0 (low-RE146), and 20.0 (medium-RE146). Taking into account that total soluble solids increased from 78 (hot liquor before moulding) to more than of 82 brix degrees (final candy after drying), the incorporation of RE to the hot liquor at 80 °C together with the heat-sensitive ingredients during the emulsion stage meant that practically all the added rosmarinic acid was retained. At the addition doses used and taking into account product dehydration, RE-enriched candies may contain up to 0.038 mg rosemary polyphenols per g product.

The results of the physical-chemical assessment are shown in Table 4. The pH values of the candies were similar (*p *> 0.05) for all treatments, meaning that the addition of RE did not affect the acidifying levels, which resulted from adding citric and lactic acids. The moisture content was slightly higher (*p* < 0.001) in the medium-RE74 and RE146 candies, meaning that dry RE powder contributed to the retention of a small quantity of water, while not modifying (*p* > 0.05) the total soluble solids and water activity values. By contrast, addition of RE led to some changes in the CIELab colour of candy. The values of lightness, redness and yellowness were not affected (*p* > 0.05) by any treatment, except in the medium-RE74 candies, which had slightly higher redness (*p* > 0.01). Nevertheless, none of the texture attributes measured in candies were affected (*p* < 0.05) by the addition of RE. Therefore, the RE doses used in candies were too low to produce any relevant physical changes, despite minor differences in moisture and colour.

Data for total polyphenols and the antioxidant status are shown in Table 5. The addition of RE74 or RE146 increased (*p* < 0.05) the total polyphenol content of the jelly candies. These increases were dose-dependent for RE146, while results for RE74 were similar at both the low and medium dose. As regards ingredients, low and medium doses of RE146 provided a higher level of GAE (300 and 572 µg/g, respectively) than low and medium doses of RE74 (113 and 285 µg/g, respectively), followed, in decreasing order, by strawberry flavouring, FOS, chicory inulin, carminic acid, and pork gelatine type “A”. Overall, the total GAE value resulting from the sum of all the individual ingredients was approximately twice that quantified in the jelly candy. Jelly candies showed medium OH-scavenging activity with percentages above 50% inhibition (damage to deoxyribose), although there were no differences (*p* > 0.05) between treatments. Citric acid and chicory inulin were the best OH-scavenging ingredients, followed, in decreasing order, by FOS = pork gelatine type “A” > lactic acid > stevia = strawberry flavouring > carminic acid > RE74 and RE146. OH-scavenging activity (measured as absorbance) decreased when ascorbate was omitted from the deoxyribose reaction to study the antioxidant pattern, meaning that jelly candy matrix behaved, on the whole, as a primary antioxidant. Similarly, citric acid and chicory inulin, the two ingredients with the highest OH-scavenging capacity, and, to a lesser extent, pork gelatine, stevia, carminic acid and lactic acid, showed lower absorbance than the control test, therefore, they can also be considered primary antioxidants. By contrast, FOS, strawberry flavouring and both rosemary extracts acted as secondary antioxidants. The TEAC values found in the jelly candies were low after 6 min. Medium-RE146 candies produced better TEAC results (*p* < 0.05) at 6 min (4.14 µmol/g) than the rest of the samples, while low-RE146 candies provided similar TEAC value to medium-RE74 candies. When measured after 24 h, all the TEAC values, including the lowest, had increased, and so the jelly candies could be considered as very good ABTS·-scavengers. The RE146 candies had the highest TEAC values, followed by RE74 candies and untreated candies. Assessment of the individual ingredients confirmed that both REs provided high TEAC values at 6 min (RE146 at the medium dose) and 24 h (RE74 at the medium dose and RE146 at both doses), while the remaining ingredients had very low TEAC values after 6 min and 24 h, with the exception of FOS at 24 h. Overall, adding RE enhanced the antioxidant capacity of candies measured as TEAC.

## 4. Discussion

The aqueous extraction of oil-free rosemary leaf provides deodorized extracts rich in rosmarinic acid suitable for use as possible functional ingredient in jelly candies, which is a water-soluble food matrix. Rosmarinic acid is a well-known radical scavenging molecule able to act as primary and second antioxidant, both in vitro and in food matrixes [18,32,33]. The polyphenols found in both REs are practically the same as those found in the methanolic RE [21], although with a lower proportion of lipophilic compounds, such as flavonoids and diterpenes. The two REs tested in our study came from flowering plants, the usual raw material processed by distillers, although the quantity of polyphenols extracted from RE146 was about twice that extracted from RE74. The phenolic composition of aromatic medicinal plants depends on their physiological stage (vegetative or flowering) when harvested. The RE obtained from flowering plants are generally poorer in phenolic acids and flavonoids and richer in diterpenes than those obtained from plants with mature fruit [20,21]. Seasonal differences in the polyphenol content of rosemary leaf may be caused by environmental factors. Southern Spain, for example, is particularly exposed to strong climatic variations, with wide temperature oscillations between winter and summer, which is characterized by many hours of sunshine and low rainfall. Sunstroke and dryness favours plant oxidative stress, inducing the formation of antioxidant secondary compounds, such as polyphenols, as a protection mechanism [32].

Rosemary off-flavour was the limiting sensory factor for aqueous RE to be incorporated in candies. Flavour assessment conducted with trained panellists confirmed that RE can be used at high doses without altering the fruity, acid and sweet characteristics of jelly candies, which are associated with the use of flavourings, acids and sweeteners, respectively, since other major ingredients such as inulin and FOS have low sweetening power. Surprisingly, RE, despite its bitterness, did not increase the bitterness of candies, due to the fact that stevia, a sweetener with an intense bitter aftertaste, may contribute to masking differences in bitterness between untreated and treated samples. In contrast, RE provided rosemary-herbal and astringent off-flavours to candies. As expected, consumers had more difficulties than the trained panellists in perceiving rosemary off-flavours in candies. Indeed, as mentioned, 0.26 g RE per kg raw candy can be added to commercial jelly candies without affecting their acceptance. At this addition level, the physical properties (pH, texture, brix, ash) of strawberry jelly candies containing RE remained practically unaltered, except for a minor difference in redness, which can be easily corrected by adjusting the dose of carminic acid. Some alterations in pH, colour or texture were found in gelatine jelly, which contained Chinese mulberry extract at 5 g/kg [34].

Several studies [14,16] agree that deodorized RE provides rosemary, bitter and astringent off-flavours due to the presence of phenol compounds and water-soluble plant components (e.g., chlorophylls, tannins, etc.) or essential oil residues [22,33]. Evidence of this being that RE74 and RE146 provided similar sensory results despite their differences in polyphenol content, suggesting that other compounds contributed to the off-flavours. No volatile phenols from essential oil residues were detected in any RE. Data regarding the sensory limitations of using plant phenolic extracts in candies are contradictory. Gramza-Michalowska and Regula (2007) [11] conducted a study in which different tea extracts (rich in catechins) were added at very high doses (10–15 g/kg) and found to be well accepted by consumers. Similarly, the addition of a Chinese mulberry extract at 5 g/kg did not alter jelly candy flavour and resulted in a product of good acceptance [10]. By contrast, a suitable level of taste masking was reached in antimicrobial gummy candies with added thyme, mandarin or grapefruit essential oils (at up to 2 g/kg) [35]. Most available information concerns meat and fish products containing RE. For example, Teruel et al. (2015) [17] found no taste alterations in fried chicken nuggets with added RE (containing essential oil) at 0.7 g/kg. Similar results were reported [36] using a lipophilic deodorized RE in salmon pate at 150 mg diterpenes per kg product (0.5 g RE/kg). Therefore, it is difficult to make any comparisons with other research due to the different extracts, doses, sensory methods, and products tested. A preliminary trial (data not shown) found that aqueous RE has more intense rosemary-herbal and bitter tones than lipophilic RE from the same plant material. Moreover, aqueous RE obtained in different conditions may provide different intensities of rosemary, astringent or bitter tones to fortified food. In this respect, the food matrix undoubtedly plays a decisive role in sensory tolerance to RE. Using fruity flavouring, sweeteners and acids may help to mask any rosemary off-flavours in jelly candies, which represents an advantage compared with other less intensely flavoured food.

The addition of RE to the hot liquor emulsion almost totally prevented rosmarinic acid degradation in the final product. Conventional jelly candies made with sugars and other jellifying agents are often cooked at temperatures below 100 °C, although it is possible to ensure microbial quality in an acidic product of low water activity by applying mild temperatures [9]. Jelly candy ingredients such as FOS syrup, the gelatine water solution and inulin cream, only need to be heated at 80–90 °C, while heat-sensitive ingredients, including RE, were continuously stirred in the hot liquor at 90 °C for only 10 min before moulding. As can be seen, any thermal damage to rosmarinic acid was irrelevant in this procedure. Rosmarinic acid has been seen to be resistant to culinary treatments in other enriched foods. Ou et al. (2018) [18] studied the remaining levels of different polyphenols added to fortified cookies at 0.02% (*w*:*w*). After baking at 190 °C for 10 min, rosmarinic acid had a higher recovery (73%) than trans-resveratrol (27%) and epicatechin (10%), which indicates that most of the added rosmarinic acid had not been degraded by cooking, as occurred in our study. Likewise, Skendi et al. (2019) [37] achieved a good recovery of rosmarinic acid in wheat bread fortified with oregano, thyme, and satureja and baked at 210 °C for 23 min. Thus, rosmarinic acid was seen to be resistant to heating. Moreover, the recovery of polyphenols in jelly candies may be affected by other factors such as handling, interaction with other ingredients, particularly carbohydrates, storage conditions, etc.

As mentioned above, RE addition increased the total polyphenol content of jelly candies. GAE concentrations reflected the polyphenols quantified by HPLC and confirmed that the RE146 had a higher antioxidant potential than the RE74. However, the GAE level was only dose-dependent in the RE146 candies perhaps owing to the contribution to GAE of other ingredients. da Silva et al. (2016) [38] found GAE recovery percentages ranging from 72% to 78% in chewy candies made with acai and no added sucrose. The GAE levels found in our study for the medium-RE74 and RE146 candies (227 and 572 mg GAE/100 g, respectively) were coherent with those reported in jelly candies containing tea extracts at 10–15 g/kg (246–1256 mg GAE/100 g) [11], and in sugar candies based on polyalcohols and ascorbic acid (2 g/kg) with menthe or camomile extracts (222–408 mg GAE/100 g) [13]. Like rosemary polyphenols, other compounds present in candy ingredients containing aromatic rings (carminic acid and strawberry aromatic esters), fructose residues (FOS and inulin) or free amino acids (pork gelatine) may present some response to the Folin–Ciocalteu reagent [39,40], which should be taken into account when assessing antioxidants from GAE values in jelly candies. This underlines the interest in knowing the response of each ingredient at the doses used in food products [30]. For example, stevia powder, a product containing ent-kaurene diterpenoid glycosides [41], did not provide GAE at the very low dose used in our candies.

The deoxyribose assay revealed that rosemary polyphenols, FOS and strawberry aromatic esters act as secondary antioxidants in this sugary-acidic matrix. Hydroxyl radicals are extremely reactive in biological systems, and the deoxyribose assay is used to detect possible scavengers of OH radicals. The products of the OH attack on deoxyribose are evaluated with thiobarbituric acid [30]. As seen, several ingredients may contribute to the deoxyribose reaction in jelly candies. Citric acid and chicory inulin were seen to be the best hydroxyl radical scavengers. Inulin exhibits the best OH-scavenging activity among soluble carbohydrates, as it is a long-chain soluble polymer with many places where the reaction with OH may derive in a proton and an electron to form a water molecule [42]. Therefore, inulin can be expected to scavenge these OH radicals. Citric acid may act as a proton-carrier under redox conditions in fruit juices and probably in similar food matrixes, increasing antioxidant activity [43]. Thus, the existing acidic conditions in candies might have enhanced the OH radical scavenging effect of citric acid. On the other hand, the TEAC assay discriminated RE antioxidant activity in candies. A TEAC value can be assigned to all compounds capable of scavenging the ABTS—by comparing their scavenging capacity with that of Trolox (a water-soluble vitamin E analogue). A quantitative evaluation of the antioxidant capacity using TEAC can be used to provide a ranking order of antioxidants. The results obtained at 24 h provide a good idea of how antioxidant nutrients may act in digestive apparatus [30]. Low- and fast-acting antioxidants exist, and their TEAC values are partially dependent on the number of free phenolic hydroxyls and the type of linkage structures in the food matrix [44]. As can be seen, TEAC values increased as the concentration of polyphenols increased and were very high for the REs. However, as with GAE, the sum of TEAC provided by the separate ingredients was higher than those determined in the final product, suggesting that interactions between the ingredients may lead to a certain loss of antioxidant properties.

Gelatine-based candies show a low level of antioxidant activities which can be attributed to the presence of amino acids such as glycine and proline [39]. Furthermore, antioxidant compounds can interact with the gelling matrix in different manners, favouring (or not) their activities in gummy candies [45]. Aromatic plant extracts are known for their good antioxidant properties. Melissa waste also had a higher antioxidant capacity than lavender waste as the latter is richer in polyphenols, which are strongly responsible for their antioxidant effects in bread [33]. In another study, the addition of rosmarinic acid meant an increase in antioxidant capacity in baked cookies [18]. Likewise, the use of extracts rich in rosmarinic acid from different aromatic plants (melissa, lavender oregano, thyme and satureja) improved the antioxidant capacity in bread [33,37]. We found no specific trials for RE-enriched candies but other phenolic ingredients, including, guajava extract [46], menthe and chamomile extracts [13], and grape by-products or betanin encapsulated with nanoliposomes [6,8] showed antioxidant activities in candy matrixes. Similarly, the addition of corn concentrates at high doses (3–12 g/100 g) also enhanced the antioxidant activities in jelly candies [47], while corn fibre gum exhibited good antioxidant activity in concentration-dependent manner [48], which agrees with our results. Other ingredients have been reported as protecting antioxidants against heat in sugary products. Stevia (at 5 mg/kg) was able to increase the antioxidant capacity in marshmallows [5]. Goji fruit jelly suffers a higher loss of antioxidant capacity than its jam because the higher sugar content and lower pH of jam protect bioactive compounds from degradation [49]. In our study, the addition of a small quantity of rosemary antioxidants enhanced TEAC values in jelly candies to the levels seen in some fruits such as pear, apricot or white grape [50], which is of particular interest for a product based on carbohydrates.

## 5. Conclusions

Rosmarinic acid-rich aqueous RE can be incorporated as an antioxidant ingredient in jelly candies without affecting their consumer acceptance. The rosemary-herbal, pungent and bitter off-flavours that are sometimes associated with rosemary can be avoided at addition levels that are still able to exert antioxidant activities in candies. The required dose of RE is too low to produce physical alterations. Rosemary polyphenols interact with other ingredients of novel candies and act as secondary antioxidants. Rosmarinic acid is very resistant to cooking and is retained without suffering degradation. At the doses used, RE-enriched candies provide a modest quantity of rosemary polyphenols to the diet (up to 0.038 mg/g), which would need to be increased to improve the functional potential of this type of candy. Enrichment with plant extracts rich in polyphenols represents a natural strategy for improving (technologically and nutritionally) certain foods with a low nutritional profile, such as jelly candies, since healthy compounds can be provided to the diet of a large and heterogeneous number of consumers. Whatever the case, more exhaustive studies will be necessary to determine the stability of polyphenols during the shelf life of the candies, as well as the recommended doses and potential benefits for both product stability and human health.

## Figures and Tables

**Table 1:** Polyphenol content of rosemary extracts.

Polyphenols	RE74		RE146	
	M	SEM	M	SEM
Phenolic acids				
Salvianic acid	1.86	0.09	16.88	0.25
Caffeic acid	1.64	0.01	<LQ	
Ferulic acid	1.42	0.02	<LQ	
Rosmarinic acid	35.09	0.18	76.77	1.45
Flavonoids				
Luteolin	5.93	0.04	7.62	0.01
Luteolin-7-*O-*glucoronide	<LQ		10.77	0.15
Luteolin glucoside derivative	<LQ		11.88	0.16
Cirsimaritin	0.89	0.01	2.64	0.01
Genkwanin	0.31	0.01	3.33	0.01
Hesperidin	3.11	0.22	11.25	0.20
Diterpenes				
Carnosol	14.94	0.12	0.38	0.03
Carnosic acid	4.85	0.04	4.13	0.01
2-O-methylcarnosic acid	2.17	0.07	<LQ	
7-methyl-rosmanol	1.72	0.02	<LQ	
Total content	73.93	73.93	145.65	145.65

Abbreviations: RE74 and RE146: rosemary extract of different polyphenol content; M: Mean; SEM: Standard Error of Mean. All values expressed as mg/g.

**Table 2:** Effects of adding rosemary extracts on jelly flavour scoring.

Attribute	ER Dose	RE74 Candies		RE146 Candies			Effects		
							RE	D	RE x D
		M		M		SEM	*p*-values		
Strawberry	Untreated	2.56		2.68		0.11	NS	NS	NS
	Low	2.63		2.58					
	Medium	2.52		2.59					
	High	2.50		2.69					
Sweet	Untreated	2.44		2.63		0.12	NS	NS	NS
	Low	2.59		2.56					
	Medium	2.46		2.57					
	High	2.43		2.77					
Acid	Untreated	2.19		2.40		0.16	NS	NS	NS
	Low	2.33		2.32					
	Medium	2.25		2.22					
	High	2.13		2.35					
Bitter	Untreated	2.34		2.45		0.12	NS	NS	NS
	Low	2.44		2.53					
	Medium	2.55		2.44					
	High	2.38		2.36					
Astringent	Untreated	1.59	^b^	1.74	^ab^	0.14	**	**	NS
	Low	1.77	^ab^	1.98	^ab^				
	Medium	1.85	^ab^	2.14	^a^				
	High	1.88	^ab^	2.13	^a^				
Rosemary	Untreated	1.69	^c^	1.56	^c^	0.17	NS	***	NS
	Low	2.07	^bc^	1.99	^bc^				
	Medium	2.32	^ab^	2.36	^ab^				
	High	2.62	^a^	2.70	^a^				

Abbreviations: RE74 and RE146: rosemary extract of different polyphenol contents; M: Mean; SEM: Standard Error of Mean; *p*-values: probability; RE: Rosemary Extract: D: Dose. RE addition levels (g/kg raw jelly): Low (0.13; Medium (0.26); and High (0.52). ^a, b, c^ RE x D effects for *** *p* < 0.001; ** *p* < 0.01; ^NS^ *p* > 0.05.

**Table 3:** Flavour identification of jelly candies by consumers in a triangle test.

	RE74 Candies			RE146 Candies		
	Consumer Trials	Correct Identifications	*p*-Value	Consumer Trials	Correct Identifications	*p*-Value
Untreated vs. Low dose	100	30	NS	100	30	NS
Untreated vs. Medium dose	100	38	NS	100	33	NS

Abbreviations: RE74 and RE146: rosemary extract of different polyphenol contents; *p*-values: probability; NS: not significant. 42 or more correct identifications are required to be significant (*p* < 0.05) (ISO 4120:2004).

**Table 4:** Effects of adding rosemary extract on the physical-chemical properties of jelly candies.

		RE74 Candies		RE146 Candies			Effects		
Property	ER Dose						RE	D	RExD
		M		M		SEM	*p*-Values		
pH	Untreated	3.03		2.96		0.02	NS	NS	NS
	Low	3.06		2.98					
	Medium	3.01		2.97					
Soluble solids	Untreated	82.48		83.80		1.38	NS	NS	NS
Brix	Low	82.69		80.99					
	Medium	84.00		82.49					
Moisture	Untreated	21.60	^b^	21.83	^b^	0.17	NS	***	*
g/100 g	Low	22.55	^a^	22.06	^ab^				
	Medium	22.42	^a^	22.42	^a^				
Water activity	Untreated	0.66		0.66		0.01	NS	NS	NS
	Low	0.67		0.65					
	Medium	0.67		0.64					
Ash	Untreated	0.19		0.18		0.00	NS	NS	NS
g/100 g	Low	0.19		0.18					
	Medium	0.19		0.18					
Lightness	Untreated	26.06		26.13		0.64	NS	NS	NS
CIE units	Low	25.76		26.08					
	Medium	26.41		26.12					
Redness	Untreated	19.71	^ab^	18.72	^ab^	0.65	**	NS	NS
CIE units	Low	20.31	^ab^	18.49	^b^				
	Medium	20.75	^a^	18.26	^b^				
Yellowness	Untreated	0.63		0.68		0.04	NS	NS	NS
CIE units	Low	0.63		0.68					
	Medium	0.64		0.66					
Hardness	Untreated	11.22		11.23		0.15	NS	NS	NS
N	Low	11.35		11.33					
	Medium	11.54		11.53					
Springiness	Untreated	4.44		4.44		0.05	NS	NS	NS
mm	Low	4.41		4.42					
	Medium	4.42		4.41					
Cohesiveness	Untreated	0.78		0.77		0.13	NS	NS	NS
No units	Low	0.76		0.76					
	Medium	0.76		0.77					
Chewiness	Untreated	39.39		39.43		0.48	NS	NS	NS
N x mm	Low	39.47		39.50					
	Medium	39.51		39.56					

Abbreviations: RE74 and RE146: rosemary extract of different polyphenol content; M: Mean; SEM: Standard Error of Mean; *p*-values: probability; RE: Rosemary Extract: D: Dose. ^a, b^ RE x D effects for *** *p* < 0.001; ** *p* < 0.01; * *p* < 0.05; ^NS^ *p* > 0.05.

**Table 5:** Effects of adding rosemary extract on the polyphenol content and antioxidant status of jelly candies and their ingredients.

Candies & Ingredients	Total Polyphenols		Damage to Deoxyribose			TEAC			
			MR+DR	MR + DR	Omit ASC	6 min		24 h	
	μg/g		A_532nm_	% Inhibition	A_532nm_	μmol Trolox/g			
Control			1.39		0.32				
RE74 candies									
Untreated	190.48	^c^	0.63	54.40	0.23	1.58	^e^	6.78	^bc^
Low	261.44	^b^	0.61	55.80	0.21	2.22	^cd^	8.40	^b^
Medium	283.24	^b^	0.64	53.97	0.20	2.73	^bc^	8.07	^bc^
RE146 candies									
Untreated	197.23	^c^	0.63	54.33	0.11	1.77	^de^	6.37	^c^
Low	273.11	^b^	0.62	54.84	0.06	3.22	^b^	10.51	^a^
Medium	410.79	^a^	0.59	59.98	0.05	4.14	^a^	11.03	^a^
SEM	12.38			3.25		0.17		0.54	
P-values									
RE-Effect	***			NS		***		***	
D-Effect	***			NS		***		***	
RE x D Effect	***			NS		**		**	
Ingredients ^(1)^									
Fructooligosaccharides	59.87		0.61	55.90	0.73	1,73		10.39	
Pork gelatine type ¨A¨	23.12		0.65	53.57	0.15	1.68		5.73	
Chicory inulin	38.88		0.38	72.42	0.20	0.35		1.76	
Stevia	-		0.94	32.69	0.14	0.61		0.73	
Carminic Acid	51.74		1.19	14.69	0.20	1.12		3.99	
Strawberry flavouring	149.58		0.94	32.86	0.44	1.12		2.50	
Acid lactic	-		0.79	43.16	0.12	0.25		0.54	
Acid citric	-		0.21	84.92	0.12	0.61		0.70	
Sodium citrate	-		1.92	-	0.48	0.48		0.67	
RE74 - Low	113.48		1.29	6.80	0.34	4.10		7.79	
RE74 - Medium	285.29		1.27	8.33	0.38	7.54		14.14	
RE146 - Low	300.19		1.26	9.61	0.40	7.76		14.35	
RE146 - Medium	571.93		1.30	4.18	0.47	12.76		14.96	
Standards									
Propyl gallate	-		1.82	-	0.61	19.75		20.29	

Abbreviations: RE74 and RE146: rosemary extract of different polyphenol contents; M: Mean; SEM: Standard Error of Mean; *p*-values: probability; RE: Rosemary Extract: D: Dose; MR+DR Added to reaction to mixture at the same concentration level). ASC: ascorbate TEAC: Trolox Equivalent Antioxidant Capacity. (-) No inhibition. ^(1)^ Analysed at the concentrations used in raw jelly candies. ^a, b, c, d^ RE x D effects for *** *p* < 0.001; ** *p* < 0.01; ^NS^ *p* > 0.05.
